# A computational method for designing diverse linear epitopes including citrullinated peptides with desired binding affinities to intravenous immunoglobulin

**DOI:** 10.1186/s12859-016-1008-7

**Published:** 2016-04-08

**Authors:** Rob Patro, Raquel Norel, Robert J. Prill, Julio Saez-Rodriguez, Peter Lorenz, Felix Steinbeck, Bjoern Ziems, Mitja Luštrek, Nicola Barbarini, Alessandra Tiengo, Riccardo Bellazzi, Hans-Jürgen Thiesen, Gustavo Stolovitzky, Carl Kingsford

**Affiliations:** Department of Computer Science, Stony Brook, NY, USA; IBM T.J. Watson Research Center, Yorktown Heights, NY, USA; European Molecular Biology Laboratory, European Bioinformatics Institute (EMBL-EBI), Hinxton, Cambridgeshire, UK; Institute of Immunology, University of Rostock, Rostock, Germany; Gesellschaft für Individualisierte Medizin (IndyMed) mbH, Rostock, Germany; Department of Intelligent Systems, Jožef Stefan Institute, Ljubljana, Slovenia; Department of Electrical, Computer and Biomedical Engineering, University of Pavia, Pavia, Italy; Computational Biology Department, School of Computer Science, Carnegie Mellon University, Pittsburgh, PA, USA

**Keywords:** Protein binding, Machine learning, Antibodies, Protein design

## Abstract

**Background:**

Understanding the interactions between antibodies and the linear epitopes that they recognize is an important task in the study of immunological diseases. We present a novel computational method for the design of linear epitopes of specified binding affinity to Intravenous Immunoglobulin (IVIg).

**Results:**

We show that the method, called Pythia-design can accurately design peptides with both high-binding affinity and low binding affinity to IVIg. To show this, we experimentally constructed and tested the computationally constructed designs. We further show experimentally that these designed peptides are more accurate that those produced by a recent method for the same task. Pythia-design is based on combining random walks with an ensemble of probabilistic support vector machines (SVM) classifiers, and we show that it produces a diverse set of designed peptides, an important property to develop robust sets of candidates for construction. We show that by combining Pythia-design and the method of (PloS ONE 6(8):23616, 2011), we are able to produce an even more accurate collection of designed peptides. Analysis of the experimental validation of Pythia-design peptides indicates that binding of IVIg is favored by epitopes that contain trypthophan and cysteine.

**Conclusions:**

Our method, Pythia-design, is able to generate a diverse set of binding and non-binding peptides, and its designs have been experimentally shown to be accurate.

**Electronic supplementary material:**

The online version of this article (doi:10.1186/s12859-016-1008-7) contains supplementary material, which is available to authorized users.

## Background

Antibody-protein interactions play a major role in infectious diseases, autoimmune diseases, oncology, vaccination and therapeutic interventions. Antibodies present in human blood interact with antigens (i.e. protein/polypeptides epitopes) with different affinities and in a sequence- and structure-specific manner. When studying protein-antibody interactions, two types of epitopes are to be distinguished: (i) conformational and (ii) linear epitopes. In this study we focus on linear epitopes; see a recent review [1] for a discussion of conformational epitopes. All potential linear epitopes of a protein can be represented by short peptides derived from the primary amino acid sequence.

The binding site of an epitope covered by an antibody typically includes a minimal stretch of 8 to 9 amino acids. If peptides of 15 amino acids in length are incubated with one specific antibody, that antibody will bind to its epitope independently of the physical position of the binding motif within the peptide. Motifs running from position 1 to position 9 up to motifs running from position 7 to position 15 would be possible. This uncertainty results in difficulties for determining consensus binding sites as well as meaningful position weight matrices (PWM). Individual amino acids within epitope binding sites may have different impact on antibody recognition not only due to the nature of amino acids involved in binding (physicochemical properties) but also because of the specific position of the amino acid within the whole peptide sequence (context).

Here, we present a method, Pythia-design, for designing novel peptides with a desired binding affinity (either high or low). This method is built upon a successful, novel discriminative classifier called Pythia (Section “Discriminative classifier for predicting binding and non-binding epitopes”) that can accurately label a given peptide as either a high- or low-affinity binder. To test the quality of the designs that Pythia-design produces, we experimentally constructed our designed peptides (and those of a recent alternative method, Barbarini et al. [2], designed for the same task) and tested their binding affinity. We show that Pythia-design more accurately designs such peptides than Barbarini et al. [2]. We further show that Pythia-design produces a more diverse set of designed peptides, which is important for generating a varied set for experimental construction. Finally, we show that the two methods of Pythia-design and Barbarini et al. [2] can be combined, exploiting the relative strengths of both, to achieve even higher accuracy in epitope design.

While there is less prior work on epitope design (e.g. [2, 3]), much previous work has focused on the task of predicting binding affinity of a given peptide to various target molecules [4], e.g. antibodies [5], to MHC class I and class II complexes alone or in concert with T cell receptor binding [6–8]. Machine learning classifiers such as artificial neural networks [9, 10], hidden Markov models [11], and support vector machines [12] and other approaches have been explored in tackling the problem of predicting Human Leukocyte Antigen (HLA) binding peptides [13, 14]. Much work has also focused on the prediction of T-cell and B-cell binding peptides [15–26]. Zhao et al. [16] explore various classifiers to predict peptide T-cell binding. Using a 10-dimensional feature vector to represent each amino acid, they discover that SVMs provide the best classification performance in their task. Huang and Dai [17] also explore the classification of peptide binding to T-cells using a support vector machine classifier. They present a novel peptide feature based on combining a 20-dimensional indicator vector with amino acid similarity information encoded by the BLOSUM50 [27] matrix. Nanni and Lumini [28] introduced the MppS system that relies on an ensemble of support vector machines, trained on various physicochemical properties, to classify peptide binding to HIV-protease and T-cells. They use sequential floating forward selection [29] to select a subset of features, and combine the individual classifier predictions using the max rule [30]. More recently, Nanni and Lumini [31] have explored the use of a novel peptide-encoding scheme that relies on the use of nonlinear dimensionality reduction to extract the information encoded across a large number of physicochemical properties. They demonstrate that this novel feature representation, when used in conjunction with a support vector machine classifier, exhibits state-of-the-art performance in predicting peptide T-cell binding. Wang et al. [32] also showed that combining multiple classifiers using a consensus approach improved the classification of MHC class II peptide binding predictions. Others [33] have used motif mining for MHC I and MHC II peptides. Recently, a flexible T cell receptor docking algorithm achieved near-native predictions for 80 % of the TCR/pMHC cases [8]. Zhang et al. [18] use 3D features and a random forest classifier to predict B-cell epitopes. Lin et al. [19] provide a method for B-cell epitope prediction that exploits phylogenetic information. Yao et al. [34] introduce an SVM approach called SVMTriP for B-cell linear epitope prediction. Yao et al. [1] compare various methods to find conformational epitopes of B-cells. El-Manzalawy et al. [23, 35] used support vector machines in combination with a subsequence kernel reaching a AUC of 0.812 and an accuracy of 73.37 % to predict peptide/epitope-antibody-binding [5].

Our novel discriminating classifier upon which our design method is based uses an ensemble of support vector machines (SVMs) to classify design candidates. This classifier is broadly similar to that of Nanni and Lumini [28]. However, we use probabilistic SVMs with Platt’s extension [36], along with a different set of features. In addition, no other previous work deals with such a wide variety of paratopes — the regions of antibodies which recognize antigens — as is found in intravenous immunoglobulin fractions, as we do here.

As our experiments below show, a diverse set of IVIg-binding peptides can be computationally designed using Pythia-design with high accuracy. In addition, our random-walk strategy for ensuring diversity is general and could be applied to any accurate discriminative classifier. These computational techniques, and the collection of designed and validated IVIg binders and non-binders, will be useful both for gaining a more thorough understanding of IVIg binding properties and the diversity of possible epitopes.

## Methods

### Overview

The Pythia-design method has two main parts. The first part is a machine learning classifier that is trained to predict whether a given peptide is a high-affinity or low-affinity binder. We designed this classifier, which we refer to as Pythia, to use an ensemble of probabilistic support vector machines (Section “Discriminative classifier for predicting binding and non-binding epitopes”) trained on various sequence, chemical, and structural features. The features used in the classifiers are described in (Section “Features used in the classifiers”). For features where we did not compute the kernel directly, we used a radial basis function kernel. The second part of Pythia-design is a method for using random walks to generate candidate peptides with novel sequences to feed into the classifier. This is described in Section “Generating novel peptides that bind to IVIg”.

### Discriminative classifier for predicting binding and non-binding epitopes

We here describe a method for predicting whether a given peptide (epitope) is going to be a low-affinity or high-affinity binder. Motivated by the success of previous work in various protein-related prediction tasks [12, 16, 28, 31, 37], we use Support Vector Machines (SVM) as our classifiers within the ensemble. We trained these SVMs via the libsvm software [38]. For all features, the optimal SVM parameters were discovered via a grid search (using libsvm’s ‘grid.py’ script) and 5-fold cross-validation. The parameters were selected based entirely on a training set without the availability of the subsequent testing set, which was held hidden from method developers until after predictions were made. For a given SVM model, the cross-validation accuracy for the optimal set of parameters is used as a weight to combine the corresponding model’s predictions with the others from the ensemble.

During standard SVM classification, instances are assigned a hard label, as belonging to the negative (low-binding) or positive class (high-binding), denoted by $\mathcal {C}^{-}$ and $\mathcal {C}^{+}$ respectively. Such a hard labeling poses no problem when only a single classifier is used to label test data. However, when an ensemble of classifiers is used, it is useful to have extra information about the degree to which the label assigned by each individual classifier should be trusted.

For this reason, we chose to use Platt’s extension [36], which provides probabilistic outputs for a support vector machine’s classifications. Instead of receiving a 0/1 label, each instance is given an *a posteriori* estimate of the probability with which it belongs to the positive class. Thus, we expect that instances that clearly belong to the negative class will be given a value close to 0, while instances that belong to the positive class will be given values close to 1.

Having a probabilistic interpretation of the classification for data instances makes it possible to combine the output of different classifiers. We used a variant of the sum rule, where the predictions of the individual classifiers are summed and normalized to yield the prediction of the ensemble. Specifically, the prediction of the ensemble for a particular instance **x**^*i*^ was computed using

(1) $$ p_{+}^{\text{ens}}\left(\mathbf{x}^{i}\right) = \frac{1}{A} \sum_{j=1}^{M} a^{j} p_{+}^{j}\left(\mathbf{x}^{i}\right),   $$

where **x**^*i*^ is a feature vector representing the *i*th peptide, constructed using some subset of the features described in Section “Features used in the classifiers” (*M*=7), $p_{+}^{j}\left (\mathbf {x}^{i}\right)$ is the *a posteriori* probability output by classifier *j* that the peptide with features **x**^*i*^ is a high-affinity binder, and *a*^*j*^ is classifier *j*’s cross-validation accuracy. *A* is a normalization factor equal to $\sum _{j=0}^{M} a^{j}$. We can then take $p_{+}^{\text {ens}}$ to be the probability with which the ensemble predicts **x**^*i*^ to belong to the positive class, or we can use it to obtain a discrete class prediction with the decision rule:

$$\mathbf{x}^{i} \in\left\{ \begin{array}{ll} \mathcal{C}^{+} & \text{if}~p^{\text{ens}}_{+}\left(\mathbf{x}^{i}\right) \ge \tau,\\ \mathcal{C}^{-} & \text{otherwise.} \end{array}\right.  $$

In our experiments, we set *τ*=0.5, but other values may be reasonable. In fact, one may even learn the value of *τ* which yields the best performance by using a held-out subset of the training data, though we do not explore that here.

Each SVM model will yield a prediction for each peptide in the testing set. We combined the predictions for all of the classifiers in the ensemble using a variation on the approach presented by Nanni and Lumini [28], which is itself an extension of the sum-rule. We normalized the predictions for each classifier to have a standard deviation of 1. Next, we combined the predictions from each of the *j* classifiers according to Eq. 1. By sorting the peptides in the testing set according to this value, we can produce a rank ordered list of the peptides in order of the likelihood that they belong to the positive (high binding affinity) class.

### Features used in the classifiers

#### Numerically encoded sequence features

There are two distinct types of sequence features that we encode numerically. First, we used a simple variation on the peptide encoding scheme presented by Huang and Dai [17]. We encoded each amino acid in the peptide by replacing its single letter code with its corresponding row in the BLOSUM50 matrix. The BLOSUM50 matrix contains empirically derived log-odds scores that encode the frequency of different amino acid substitutions and is commonly used to measure the similarity between different amino acids. Let the peptide of length *d* be given as **p**=(*a*_0_,*a*_1_,…,*a*_*d*_), where *a*_*i*_ is the amino acid in the *i*^th^ position of the peptide. Further, let row(*a*) map the amino acid *a* to its corresponding row in the BLOSUM50 matrix. We encoded the peptide as enc(**p**)=(row*a*_0_,row*a*_1_,…,row*a*_*d*_). For the length *d* peptide **p**, enc(**p**) will be a 20*d* dimensional feature vector. In addition to BLOSUM50, we use the same type of encoding with matrices **nlf** and **sa** introduced by Nanni and Lumini [31]. These matrices are derived by performing dimensionality reduction on a large, rectangular (i.e. 20×*k* with *k*≫20) matrix where each row corresponds to an amino acid and each column to some physicochemical property. The goal of the dimensionality reduction is to decorrelate the physicochemical properties, reducing the column space of the matrix significantly. The **nlf** matrix is a 20×18 matrix obtained using a nonlinear fisher transform, while the **sa** matrix is a 20×10 matrix obtained using a combination of clustering and principal component analysis.

The second type of sequence feature that we encode numerically involves various physicochemical properties of the constituent amino acids of each peptide. We analyze the amino acid properties present in the Amino Acid Index (AAIndex) [39]. Each AAIndex property provides a mapping from each of the 20 amino acids to a numerical scale measuring some physicochemical attribute (e.g. hydrophobicity, antigenicity). The AAIndex listed 544 different amino acid properties. We use an approach based on a sliding window and histograms to turn each AAIndex property into a numerical feature vector for a peptide. Consider a single AAIndex property AAI^*j*^, and let AAI^*j*^(*a*) represent the numerical value to which the amino acid *a* is mapped under AAIndex property *j*. To form a representation for the entire peptide **p** under the property AAI^*j*^, we used a window of length *w* sliding across the peptide to produce a (*d*−*w*+1)-dimensional vector where entry *i* in this vector is the average value of the AAIndex property over the window starting at position *i*. By varying *w*, we can change the coarseness of this representation. Through a process of experimenting with different values of *w* for this classification task (using only training data), we computed these features for *w*∈ [ 3,5].

#### String kernel features

String kernels are used to evaluate the sequence similarity between peptides. There are many different varieties of string kernels, ranging from the somewhat simple *k*-spectrum kernel, which essentially counts the occurrence of all length *k* substrings in each peptide, to the more complex substring-mismatch kernel [40], which considers all shared subsequences between two peptides, allowing for gaps and mismatches.We use the *k*-spectrum string kernel [37] for *k*=3,4,5,6, the SSSK kernel [41] with parameter *d*=6, and the bounded range substring kernel [42] with parameter *r*=8. The output of each of these methods is a matrix, known as the kernel matrix, in which the entry at row *i* and column *j* is the result of the kernel evaluation between peptides *i* and *j*. To train a SVM model for each of these string kernels, we simply compute the kernel matrix, and then make use of the ability of libsvm to train a model using a precomputed kernel.

#### Structure features

We use rigid docking to estimate the binding affinity between IVIg and a candidate peptide. We computed a hypothesized 3-dimensional structure for each peptide using the Biochemical Algorithms Library (BALL) [43]. We built a starting model for each peptide **p** by positioning the side chains for each amino acid by choosing the most frequently occurring rotamer position from a rotamer library. We then optimized this initial structure by performing an energy minimization using the AMBER [44] force field. This relaxes the structure until a (possibly local) energy minimum is achieved.

We also obtained an experimentally measured 3D structure for IgG1 [45], the most prevalent class of IgG antibody present in intravenous immunoglobulin. We measure the conformational complementarity of each of our hypothesized peptide structures with the immunoglobulin structure. To compute this complementarity, we performed a protein-protein docking simulation for each of the constructed peptides against IgG1 using the ZDock software [46]. Each ZDock run produces a list of the 2000 top-ranked (according to ZDock’s criteria) docking predictions for each peptide. The ZDock score provides a measure of the complementarity of the peptide and immunoglobulin conformation in the docking region and is used as a proxy for the overall quality of the docking. For each peptide, we formed a histogram from the 2000 ZDock scores, and use this histogram as a feature vector with which to train the SVM model. Intuitively, we expect peptides whose ZDock score distributions are skewed toward high scores to have better shape complementarity and, therefore, to be more likely binders than peptides whose ZDock score distributions are skewed toward low scores.

### Generating novel peptides that bind to IVIg

Pythia-design builds upon the classifier described above by first generating many peptide sequences and then assigning them a reactivity category high (H), medium (M), or low (L) according to the predictions of our classifier. We generated the *de novo* sequences using a sampling approach that corresponds to a seeded random walk in sequence space. To obtain a sequence for reactivity class *C*, we choose a random seed sequence *s*∈*C* (such as *H*) from the training set, and randomly mutate its constituent amino acids until it adheres to several required sequence diversity rules, in order to ensure that the novel peptide sequences that are generated are sufficiently different from those in the training set. Specifically, the designed sequences predicted to be in the high- or low-reactivity category could not share any 4-mer, or exhibit a sequence identity of greater than 6 amino acids in any subsequence of length 11, with any sequence in the same reactivity category in the training set. Further, the peptides generated by the Pythia-design method were required to adhere to the same set of constraints with respect to the peptides in the original experiment’s testing set.

We seeded our peptide generator with 6000 sequences from the training set — 3000 sequences with the highest experimentally measured reactivity and 3000 sequences with the lowest experimentally measured reactivity. Running the random walks then produced 6000 candidate peptides that we classified using Pythia and sorted according to their probability of belonging to the positive class. There were 2468 peptides with an probability greater than or equal to 0.5, and 3542 with an probability less than 0.5. The 1500 sequences with the highest probabilities were predicted belong to the high-affinity class *H*, while the 1500 sequences with the lowest probabilities were predicted to belong to the low-affinity class *L*. The remaining 3000 peptides, with probabilities closest to 0.5, were predicted to belong to the medium-affinity class *M*.

### Training and testing sets of peptides for classifier

To train and test our discriminative classifier, we used the data set of Luštrek et al. [5] (included as Additional file 1: Table S1), in which 75,534 peptides were incubated with commercially available intravenous immunoglobulin (IVIg) fractions, which was originally presented as part of the DREAM5 challenge 1 (https://www.synapse.org/#!Synapse:syn2820433/wiki/71017). IVIg is a mixture of naturally occurring human antibodies isolated from up to 100,000 healthy individuals. From this dataset, high-confidence negative and positive pools of recognized peptides were determined based on epitope-antibody-reactivities (EAR) of more than 75,000 different peptides subjected to a huge number of structurally different antibodies present in IVIg. (See [5] as well.) The training and test datasets for the discriminative classifier were assembled from these peptide pools (Additional file 2: Table S2 and Additional file 3: Table S3). From the collection of all the peptides incubated with human IVIg, a pool of 6,841 epitope-containing peptide sequences reactive with human immunoglobulins (signal intensity >10,000) was experimentally identified. This was called the positive set. From the same original collection of peptides 20,437 peptides were identified that showed no antibody binding activity in any of the triplicate assays (signal intensity <1,000). This peptide set was called the negative set. The training set was formed by random sampling of 3,420 peptides from the positive set and 10,218 peptides from the negative set. The training set thus created contained 13,638 peptides and their respective binding reactivities. The test set was created by joining together the remaining 3,421 peptides from the positive set and the remaining 10,219 peptides from the negative set, for a total of 13,640 peptides.

### Selection of designed peptides for experimental validation

Pythia-design was used to generate 1500 peptides predicted to be reactive (high binding affinity) as well as 1500 predicted not to be reactive. The method of Barbarini et al. [2] was also used to generate 1100 peptides of each class. To select a subset to experimentally construct and validate, the designed peptides were re-categorized by selecting 400 high binders and 200 non-binders as follows. A stratified instead of a randomized sampling procedure was chosen in particular to investigate whether the designed peptides are robust to existing classification methods [5] as well as position weight matrix (PWM) analysis. The initial peptide set were subgrouped by using PWM and SVM analyses as described in [5]; see categories in legend of Additional file 4: Table S4 (column F). Categories (*n*=8) were determined from a linear scale representing PWM and Lustrek classification scores of the original data sets (Pythia: *n*=1500; Pavia: *n*=1100) from minmax sampling of the classifiers was used. The stratified quota sampling was restricted to 50 peptide sequences in case of binding peptides (in case of non-binders *N*=25). In total, 400 peptides predicted to be bound and 200 predicted not being bound by IVIg were taken from each of Pythia-design and Barbarini et al. [2] and subjected to experimental testing.

## Results

### Overview

The test set of peptides was withheld from the algorithm designers until after the algorithm was finalized. Only the training set was used in the design and initial evaluation of the Pythia classifier. Once finalized, Pythia was then evaluated on the held-out test set of peptides to validate the classifier component of Pythia-design. Finally, Pythia-design was used to generate a collection of new likely binder and non-binder peptides, a subset of which were then experimentally validated.

### Validation of the Pythia affinity classifier

Although our goal is to produce a peptide design method, we first validate that our peptide classifier is accurate. This classifier is based on an ensemble of learners that aggregates the prediction of many individual classifiers, each of which was trained on a set of features. Analyzing the AUC for the individual classifiers in the Pythia ensemble (Table 1) on the test peptides, we observe that many of the classifiers show similar performance, with the exception of the structural classifier which displays significantly lower classification performance. The ensemble, however, yields superior performance compared to any of its constituent classifiers with an AUROC of 0.893. At any given false positive rate, the ensemble classifier will obtain a higher true positive rate than any of the other classifiers. The precision-recall curve presents a related view of classifier performance to the ROC curve. It measures how the precision changes as the recall is increased. For very small recall values (i.e. recall ≤0.1), the sparse spatial sample and k-spectrum string kernels yield the best (and very similar) precision. However, for the vast majority of recall values, the ensemble classifier yields the highest precision. Just as was the case with the ROC curves, the ensemble again achieved the maximum area under the PR curve. While the AUPRs were generally lower than the AUROCs, we did observe that the benefit of the ensemble was larger with respect to the PR curves than the ROC curves.

**Table 1:** Performance of the various classifiers used within the Pythia method

Features	AUROC	AUPR	*Δ*AUROC	*Δ*AUPR
k-spectrum	0.85	0.70	−0.043	−0.072
Sparse Spatial Sample	0.87	0.73	−0.023	−0.042
Nonlinear Fisher Mat.	0.86	0.69	−0.024	−0.082
Statistical Analysis Mat.	0.85	0.67	−0.025	−0.102
BLOSUM Encoding	0.86	0.70	−0.024	−0.072
Local Composition^a^	0.88	0.74	−0.013	−0.032
Structure	0.74	0.53	−0.153	−0.242
Ensemble	**0.89**	**0.77**		

^a^the best single classifier under both the AUROC and AUPR metrics

Boldface indicates the best solution

Because the test and training sets of peptides were chosen randomly, it is possible that overlapping or shared sequences between the test and training set partially leads to this high performance. Nevertheless, our ultimate aim is to design novel peptides, and this analysis suggests that Pythia has reasonable performance identifying low- and high-affinity binding peptides.

### Accuracy of predicted affinity of computationally designed peptides

Pythia-design was used to generate a number of probable high-affinity binding peptides (*H*), probable low-affinity binding peptides (*L*), and medium-affinity binding peptides (*M*). In addition, the method of Barbarini et al. [2] was used to generate the same number of peptides. A subset of designed peptides of both the Pythia-design and Barbarini et al. [2] methods were experimentally constructed (Section “Selection of designed peptides for experimental validation”). The binding affinities of these designed peptides were then experimentally measured. Additional file 4: Table S4 gives the designed peptides and their measured affinities.

**Fig. 1:**
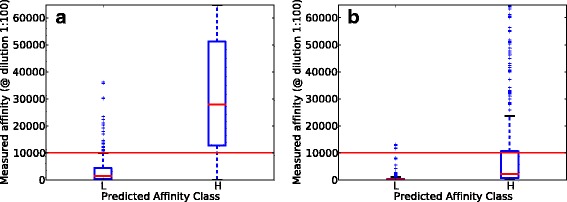
Quality of designed peptides from two approaches. The distribution of measured affinities for the designed peptides predicted to belong to the low (L) and high (H) binding affinity classes for the (**a**) Pythia-design method, and (**b**) method of Barbarini et al. [2]. The horizontal line at 10,000 indicates the binding affinity cutoff above which a peptide is considered to have a high binding affinity. Both methods produce a statistically significant separation of high- and low- binders (*P*<0.001), but Pythia-design is much better at generating high-affinity binders

Figure 1 gives the distributions of the measured affinities for the predicted high- and low-affinity designed peptides for Pythia-design and for the Barbarini et al. [2] method. Both approaches are able to design low-binding peptides well. This is presumably the easier task as one would expect there to be many more non-binding peptides than high-affinity binders. For design of binders, the presumably more challenging problem, the predicted high-binding affinity designs of Pythia-design tended to have much higher measured binding affinities than those produced by Barbarini et al. [2].

To quantify the degree to which the methods are able to generate binders and non-binders effectively, we use the method of Ojala and Garriga [47] to compute a ***P***-value indicating the probability of the observed high- and low-binding separation. To do this, we compute the standard F1-score of the predictions, then randomly permute the labels 1000 times to get a distribution of F1-scores. For both Pythia-design and Pavia, the true F1-score is always better than the randomized score, meaning that for each method *P*<0.001. This indicates that the approaches are both truly designing peptides better than random.

**Fig. 2:**
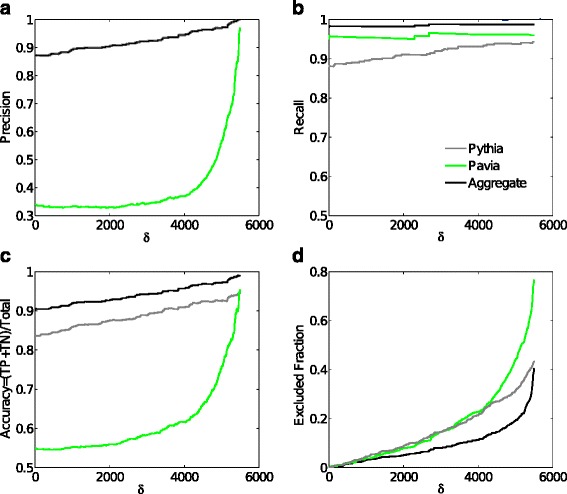
**a** Precision, **b** recall and **c** accuracy of peptide design. Performance of Pythia-design, the method of [2] (labeled Pavia), and the aggregate formed by taking the positives from Pythia-design and the negatives from [2] is shown. In (**a**), the lines for Pythia and the aggregate overlap. The x-axis *δ* is a measure of the activities from the high- and low-binding affinities (see text), and (**d**) shows the fraction of peptides excluded for a given *δ*

**Fig. 3:**
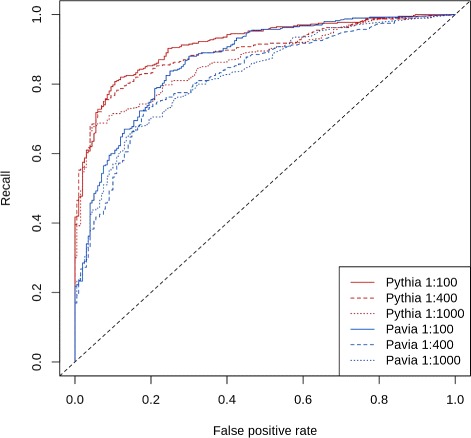
Performance of the Pythia-design and Barbarini et al. [2] method (labeled Pavia) for designing peptides with desired reactivities. ROC curves were determined from predicted peptides incubated with IVIg (5 mg/ml) diluted to 1:100, 1:400 and 1:1000, and epitope-antibody reactivities (EAR) determined as described by Lustrek et al. 2013

Using specific binding defined by a factor 10 above control measures (secondary antibody measures) plus a minimum signal of above 1000, the designed peptides were categorized as truly or falsely predicted as outlined in Additional file 4: Table S4. Using these categorizations, we computed precision, recall, and accuracy for the two methods. Since we do not have a hard threshold to define high or low affinity measurements, and such threshold cannot be detected from the distribution of measurements itself, we used a two-valued cutoff centered at 5500, the middle of the excluded range of intensity measurements. The cutoff was chosen to be 5500±*δ*, (0≤*δ*≤5449), to define the high (measurements greater than 5500+*δ*) and low (measurements less than 5500−*δ*) affinity thresholds, thus leaving out of consideration those predicted peptides with measurements that fall in the “grey” zone of [5500−*δ*,5500+*δ*]. When *δ*=0, we take into account all the measurements, when *δ*=4500 we have a grey zone of peptides with signal intensities between 1000 and 10000. Figure 2 shows the performance (precision and recall) as well as the fraction of excluded peptides for both methods as well as the aggregate prediction as a function of *δ*. From Fig. 2 top-left panel, we can see that the precision of the method of Barbarini et al. [2] at *δ*=4500 is around 0.5, much lower than the precision of close to 0.95 of Pythia-design. ROC curves showing the same conclusion are presented in Fig. 3.

### Diversity of designed peptides

**Fig. 4:**
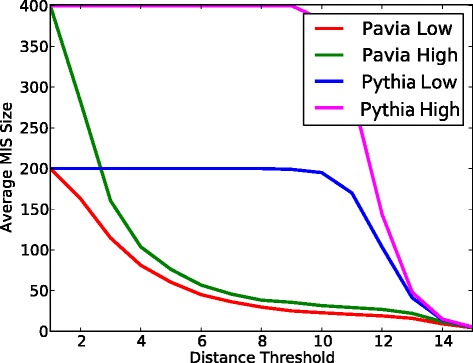
Diversity of the designed, predicted high- and low-binding affinity peptides. The sequence diversity among the Pythia-design peptides is significantly higher than the approach of [2]. The y-axis gives a measure of diversity of a set of designed peptides (see text) under a particular Hamming-distance threshold defining similar peptides (x-axis). Almost all the Pythia-designed peptides differ in at least 9 of their 15 possible positions

It is important that a design method is able to generate many diverse peptides rather than a small number of very similar peptides. This allows for a greater sampling of the space of binding peptides and allows for more effective screening based on downstream criteria. The set of peptides produced using the Pythia-design approach is much more diverse than the set generated by the Barbarini et al. [2] approach (Fig. 4). To quantify the diversity, we create a graph *G* from the set of predicted high and low binding affinity peptides, where each peptide is a vertex in *G* and two different peptides are connected by an edge if they have fewer than a specified number *c* of differences under the Hamming distance (which counts the number of disagreeing amino acids across all positions). For each vertex *v*, we compute a maximal independent set that contains *v*. An independent set is a subset of vertices such that no two are connected by an edge. This yields *n* maximal independent sets, where *n* is the order of the graph. The size of the independent set containing *v* is a measure of how dissimilar *v* is to the other designed peptides. We compute the average size of these maximal independent sets, and observe how this value changes as we vary the cutoff parameter *c* defining the edges of the graph. At a given cutoff level *c*, if the average size of the maximal independent sets is larger, then there are more independent peptides and these peptides have, by construction, *c* or more differences. Pythia-design peptides exhibit substantially more diversity (Fig. 4) than the designs of Barbarini et al. [2]. Until a distance cutoff of 9, almost all of the Pythia-design peptides (in both the high and low affinity sets) belong to a single independent set that spans the entire graph, meaning that nearly all the peptides are dissimilar in at least 9 of their positions. The designed peptides of Barbarini et al. [2], however, share a great deal of sequence similarity, representing a very dense sampling of the sequence space near only a few particular points.

### Aggregation of two strategies for constructing binding and non-binding peptides

We also created an aggregate approach for *de novo* peptide design to assess how Pythia-design complements previous methods. Pythia-design and the method of Barbarini et al. [2] take different approaches to the design problem. Pythia-design generates dissimilar random peptides and classifies them, while the Barbarini et al. [2] method extracts some motifs from clusters of positive and negative peptides and then generates new peptide sequences. The method of Barbarini et al. [2] produces a high number of peptides that, even though they were predicted positives, are actually non-reactive. On the other hand Pythia has very few non-reactive peptides in its positive set. A confusion matrix (Table 2) comparing the binding by IVIg dilutions of peptides from Pythia-design and the method of Barbarini et al. [2], showing dilutions of 1:100, 1:400, 1:1000, underline that the Pythia-design method does preferentially select peptides that show high affinity binding to IVIg.

**Table 2:** Confusion matrix for designed peptides

	Barbarini et al. [2]		Pythia-design	
	Binder	Nonbinder	Binder	Nonbinder
Bound (1:100)	261	25	387	117
Not bound (1:100)	139	175	13	83
Bound (1:400)	128	7	325	40
Not bound (1:400)	272	193	75	160
Bound (1:1000)	99	3	270	18
Not bound (1:1000)	301	197	130	182

Six hundred peptides representing 400 binders and 200 non-binders of each of Pythia-design and the method of Barbarini et al. were incubated with IVIg. The confusion matrix below indicates that the peptides selected by Pythia-design bind antibodies with higher affinity than the peptides designed by Barbarini et al.

**Fig. 5:**
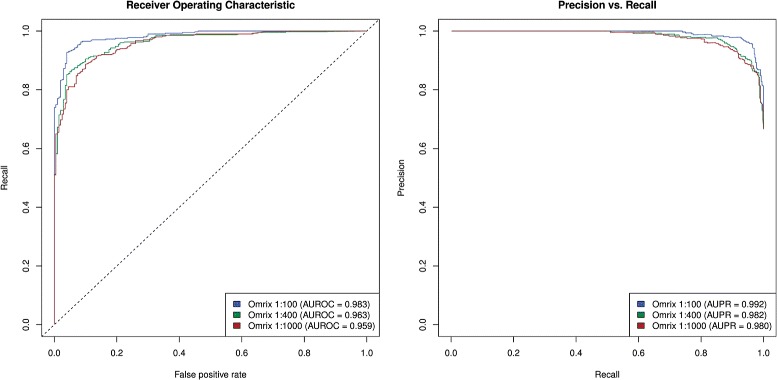
Performance of a method that combines Pythia-design and Barbarini et al. [2] (labeled Pavia)

To combine the strengths of each method, we used the strategy of Barbarini et al. [2] for generating low-affinity peptides and Pythia-design for generating high-affinity predictions. The accuracy of the aggregate method, with an AUROC of 0.959–0.983, is better than either of the two methods (Fig. 2 vs. Fig. 5). One caveat, however, is that this combined method produces a set of non-binders with lower diversity than that produced by Pythia-design in isolation.

### Inclusion of citrulline, cysteine, and tryptophan in designed peptides

**Fig. 6:**
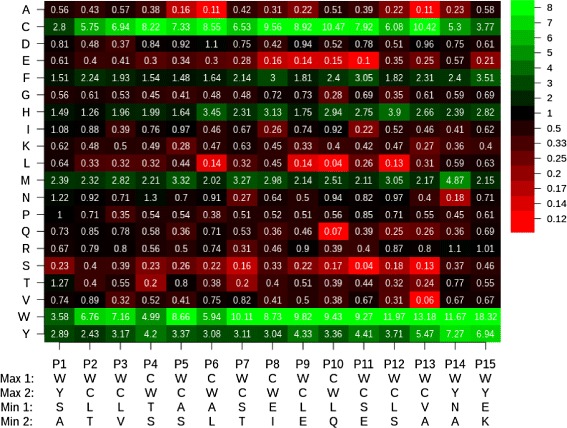
Position-specific peptide propensity within true positive Pythia-design peptides (at dilution 1:1000) divided by the PWM of the negative set of peptides. PWM segments in *red* indicate amino acids that are predicted to interfere with antibody binding. *Green* highlights amino acids that favor binding of antibodies present in IVIg. An over-representation of cysteine (C) and tryptophan (W) in all positions is seen

Pythia-design was allowed to include the citrulline amino acid (denoted Z) in its designed peptides. Many of the designed peptides included this non-standard amino acid (Table 3). Citrulline was included with high prevalence in both the tested high and low binders (318/400≈80 *%* tested high binders and 185/200≈92 *%* of tested low-binders). Since both high and low binders included them, we do not see a significant effect on binding in general with the inclusion of these amino acids. It is likely they were included with such high prevalence because such peptides are able to satisfied the imposed peptide diversity constraints. Similarly, peptides with cysteine (C) are over-represented in the Pythia-design peptides (Table 3), likely for partially similar reasons. This interpretation is further supported by the fact that C and Z were under-represented in the training and test sets used to train the Pythia discriminative classifier on which Pythia-design is based (Additional file 5: Figure S1). It is also consistent with the implementation of the classifier, which omitted features for which no training data (such as amnio acid chemical properties) were available. In particular, all of the propensity to include or exclude Z in Pythia-design comes from the string kernel and structural features extracted from the training set. Figure 6 shows that the true positive designed peptides often include tryptophan (W) and cysteine (C), which are relatively uniformly over-represented along the entire length of the designed peptides.

**Table 3:** The presence of citrulline and cysteine in the designed peptides and the training and test sets

	Total	With Z	With C	With Z and C
Training set High	3420	8	906	3
Training set Low	10218	326	1971	71
Test set High	3421	5	944	3
Test set Low	10219	356	2032	93
Pythia-design “H”	1500	1286	1302	1093
Pythia-design “M”	3000	2885	2365	2253
Pythia-design “L”	1500	1484	1044	1029
Pythia-design “H” tested	400	318	344	265
Pythia-design “L” tested	200	185	150	136
Barbarini et al. [2] “H”	1100	0	420	0
Barbarini et al. [2] “L”	1100	0	628	0
Barbarini et al. [2] “H” tested	400	0	196	0
Barbarini et al. [2] “L” tested	200	0	110	0

The prevalence of citrulline and cysteine is likely due to the fact that citrulline and cystiine were less represented in the peptides used in the training data sets, allowing these designed peptides to more easily satisfy the imposed diversity requirements

## Discussion

One caveat of epitope mapping experiments is the nature of how peptides are presented e.g. in solution or fixed to a support and/or whether the peptides are fixed amino- or carboxyterminally. The peptides that we analyzed were coupled via the aminoterminal end to glass slides [5, 48]. The nature of how peptides are presented in solution or fixed to a platform might influence the binding affinities obtained. This experimental restriction may affect their binding properties, and if peptides were bound via the carboxyterminal end, their binding affinities may change. Hecker et al. [48] have demonstrated that the epitopes found via the experimental protocol used here represent epitopes that are functional in other assay systems as well, so it is likely that the predictive methods and their results will be robust to the experimental assay. In addition, naturally the experiments reported here are specific to the IVIg sets selected. Performance on additional sets is a promising direction for future work.

Another caveat of the experimental validation performed here is the non-random selection procedure used for choosing which peptides were experimentally validated. Because stratified minmax sampling was used, the designed peptides were chosen so that they represented both high- and low- predicted binders of two other computational methods. We find post-selection that the peptides designed by Pythia-design fell relatively uniformly across these categories, so the sampling represents a mostly unbiased sampling of the designed peptides (Additional file 5: Figure S2A). The binders designed by Barbarini et al. [2] displayed more bias toward appearing in only a few sampling categories (Additional file 5: Figure S2B), and so it is possible that this has led to a bias under-representing that method’s overall performance.

Another caveat is our interpretation of the use of citrulline in our high-binding designed peptides. Although it appears that citrulline is primarily included only to increase peptide diversity, it is possible that the predicted peptides are only reactive to the subset of antibodies within the IVIg serum that bind to citrullinated peptides. In this case, the predicted results are still of interest, since they represent a high-binding set of peptides, but to this more restrictive class of antibodies.

Despite its high performance, there is room for improvement of the Pythia method for affinity binding prediction that is at the core of Pythia-design. In particular, ZDock [46], which is used to compute the structural features, considers only rigid docking of the peptides to immunoglobulin. The structural features may change significantly if we use a non-rigid docking procedure, where conformational changes in the paratope, epitope, or both are allowed. It is actually quite surprising that, using only a single IgG1 structure model and using only perfectly rigid docking, the structure-based classifier obtained such respectable performance. This indicates that improving the computation of the structural features is a promising way to increase prediction accuracy. The integration of additional structural information might guide and improve computational processes studying epitope binding. Not all immunogobulins necessarily use similar or comparable binding modes [5]. As such, knowing the structural heterogeneity of immunoglobulins found in one human individual might lead to the description of different types of epitope-antibody recognition modes [5]. Another potential source of improvement is the inclusion of phylogenetic information as was done in Lin et al. [19].

An important direction for future work is the determination in greater detail, from the SVM weights, which sequence features are particularly indicative of binding or non-binding. For a machine learning method to be reasonable, the testing and training peptides should be drawn from the same distribution, as we have done here. The peptides used to train and test the Pythia classifier were randomly chosen (in vitro) from a large set of bound and non-bound peptides, and randomly divided into testing and training. Given the very high level of performance on the random set, it is unlikely that performance is driven by any small number of sequence patterns. Further, and most importantly, the generalizability of the Pythia classifier was tested in a particularly strong way: it was used to design novel, sequence-dissimilar peptides and the accuracy on that task is very good, and provides strong evidence that the developed models are not specific to any single sequence pattern.

## Conclusion

We have provided a method, Pythia-design, for the design of peptides with specific reactivity properties (either high- or low-affinity binding), showing experimentally that the designs accurately exhibit the desired affinities. In addition to producing more accurate designs than previous approaches, Pythia-design is able to sample the space of possible designed peptides more completely, creating many dissimilar designs rather than variations on a few similar peptides. Again, combination of two dissimilar methods shows improved performance.

Understanding antibody binding patterns is crucial for understanding the immune response for many human diseases. We show that diverse sets of peptides that exhibit the desired binding properties can be computationally designed. This work moves us closer to understanding the interplay and interactions between human antibodies and the targets to which they bind.

## Additional files

Additional file 1
**Table S1.** Binding training data. Training set of measured binding affinities. This collection of peptides was used to train the Pythia classifier. (TXT 260 kb)

Additional file 2
**Table S2.** Classification Test Binders. Set of binders in the test set including computational analysis. The peptides in Column A represent the test data set, sorted by Column B. The highest measured values (MaxIVIG) are given in Column B, in Column C (mBuffer) the mean of secondary antibody control, and in Column D (mIVIG) the mean of all IVIG measures. In Column E, the sum of all computational methods are summed up whose predictions were correct as outlined in the remaining columns, where the EL-Manzalawy, Luštrek, PWM, Pythia, Barbarini et al. [2] (Pavia), and PWM2 methods are given. (XLS 1218 kb)

Additional file 3
**Table S3.** Classification Test Non-Binders. Set of non-binders in the test set including computational analysis. The peptides in Column A represent the test data set, sorted by Column B. The highest measured values (Max IVIG) are given in Column B, in Column C (mBuffer) the mean of secondary antibody control, and in Column D (mIVIG) the mean of all IVIG measures. In Column E, the sum of all computational methods are summed up whose predictions were correct as outlined in the remaining columns, as in Additional file 2: Table S2. (XLS 1218 kb)

Additional file 4
**Table S4.** Designed peptides and their experimental validation. Data set of the designed peptides including computational analysis. The peptides in Column A were further categorized as outlined in Material and Methods, see Column E and F. The Columns B, C, D show the computational assessment based on methods used in [5]. The experimental data are given in Column J, K, and L using dilutions 1:100. 1:400 and 1:1000 of IVIG purchased from Omrix. Column M gives the background values to the binding of the secondary antibody to the peptides. Columns G, H, and I outlines whether binding occurred (True) or not (False). The first Excel data sheet shows the potential binders, the second Excel sheet the non-binders. (XLS 408 kb)

Additional file 5
**Figure S1**. Distribution of amino acids within training and testing sets. **Figure S2**. Selection bias of validated peptides. (PDF 2017 kb)
