# Supplementary material for: A computational method for designing diverse linear epitopes including citrullinated peptides with desired binding affinities to intravenous immunoglobulin
Source: BMC Bioinformatics. 2016 Apr 8;17:155. doi: 10.1186/s12859-016-1008-7 (PMC4826543; doi:10.1186/s12859-016-1008-7)
Supplement: Additional file 5 — Figure S1. Distribution of amino acids within training and testing sets. Figure S2. Selection bias of validated peptides. (PDF 2017 kb) [file 12859_2016_1008_MOESM5_ESM.pdf]

Supplementary Material for “A computational method for designing diverse linear epitopes including citrullinated peptides with desired binding affinities to intravenous immunoglobulin”

Supplementary Figure 1: Distribution of amino acids within training and testing sets

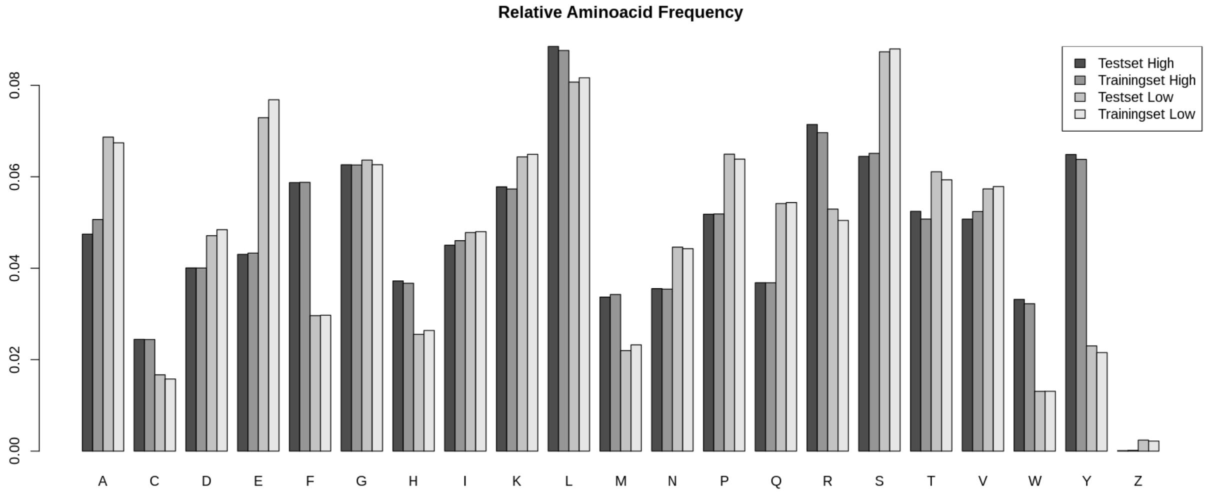

Distribution of amino acids within training and testing sets. Cystine and citrulline are under-represented within the training set used to train Pythia and the testing set used to validate the Pythia classifier. This supports the interpretation that Pythia-design has included them frequently in order to satisfy the diversity constraints.

Supplementary Figure 2: Selection bias of validated peptides

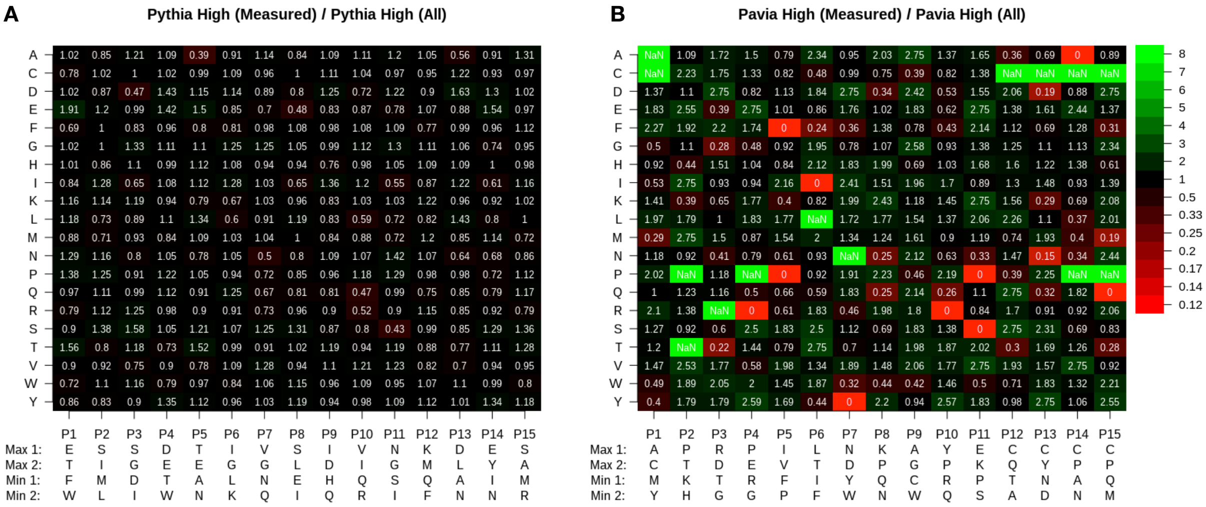

Bias in sequence features for experimentally verified peptides. (A) Position-specific amino-acid propensities comparing all Pythia-designed computationally designed peptides and the experimentally measured subset. The experimentally verified set has a similar positional sequence compensation with the entire designed set. (B) The same plot for the peptides designed by Barbarini et al. compared with the selected validated peptides. A larger sequence bias is observed. Colors as in Figure 6.
